# Dr. Romaine J. Curtiss

**Published:** 1901-01

**Authors:** 


Dr. Romaine J. Curtiss died at his residence in Joliet, Ill., November 20, 1900, after a short illness. Dr. Curtiss was born in Richland County, O., October 1, 1840. In 1862 he entered the Union Army as hospital steward of the 123d Ohio Volunteers, and in April, 1863, he was appointed a medical cadet in the regular army. Later he was appointed assistant surgeon in the navy, serving till the close of the war. He then located at Angola, Erie County, N.Y., where he practised medicine for seven years. He went to Joliet from Erie County in 1873 and has resided there since that time.

